# Mapping the symptom network of anxiety, depression, somatic symptoms and sleep disturbances in 15,642 infertile women: a retrospective cohort study identifying targets for fertility care

**DOI:** 10.3389/fpubh.2026.1884236

**Published:** 2026-08-05

**Authors:** Yan Wang, Yuyang Wang, Yue Feng, Yan Pu

**Affiliations:** 1 Department of Reproductive Medicine Nursing, West China Second University Hospital, Sichuan University, Chengdu City, China; 2 Key Laboratory of Birth Defects and Related Diseases of Women and Children (Sichuan University), Ministry of Education, Chengdu City, China; 3 Department of Gynecological Nursing, West China Second University Hospital, Sichuan University, Chengdu City, China

**Keywords:** anxiety, cohort study, depression, *in vitro* fertilization, infertility, network analysis, psychological distress

## Abstract

**Background:**

The interrelationships among common psychological distress symptoms, as well as their associations with *in vitro* fertilization–embryo transfer (IVF-ET) outcomes, have not been fully characterized. This study aimed to identify the central and bridge symptoms of the psychological distress network in infertile women, and to investigate the associations between psychological distress and IVF-ET outcomes.

**Methods:**

This retrospective cohort study included 15,642 infertile women treated between June 2020 and January 2024. Anxiety, depression, somatic symptoms, and sleep quality were assessed using validated standardized scales. A Gaussian graphical model with graphical lasso regularization was constructed to estimate the symptom network. Node centrality (expected influence, EI) and bridge centrality (bridge expected influence, BEI) were calculated, and network stability was assessed using bootstrapping. Multivariable modified Poisson regression was used to examine associations between psychological distress and IVF-ET outcomes.

**Results:**

The prevalence rates of anxiety, depression, somatic symptoms, and sleep problems were 30.1%, 34.8%, 45.1%, and 13.3%, respectively. Network analysis identified trouble relaxing (A4, EI = 1.313) and uncontrollable worry (A2, EI = 1.240) as the most highly connected central symptoms, while daytime dysfunction (SQ7, BEI = 0.887) and fatigue (D3, BEI = 0.657) emerged as the top bridge symptom. Multivariable modified Poisson regression analysis showed that better subjective sleep quality was associated with a higher likelihood of clinical pregnancy (a*RR* = 1.026, 95% CI 1.008–1.044), and sleep medication use was associated with the risk of late miscarriage (a*RR* = 0.211, 95% CI 0.052–0.861).

**Conclusion:**

Anxious cognitive and mind-body symptoms represent the most highly connected structural features of the psychological distress network in this cohort. Sleep-related problems were associated with IVF-ET outcomes, providing hypothesis-generating evidence for the potential clinical relevance of sleep assessment in infertility care.

## 1 Introduction

Infertility is a major global reproductive health concern, affecting tens of millions of people of reproductive age. The World Health Organization estimates that approximately one in six individuals experiences infertility, corresponding to a prevalence of 12.5% (1) and affecting some 48 million couples and 186 million individuals worldwide. In China, the Global Burden of Disease Study 2021 reported that female and male infertility cases account for 23.59% and 21.47% of global totals, reaching 33,795,944 and 11,909,889, respectively (2). The condition is especially prevalent in southwestern China, where rates reach 20% and, in certain areas, exceed 25% (3), increasingly affecting younger populations. The high burden of infertility is associated with substantial impairments in patients' family and social functioning (4).

*In vitro* fertilization–embryo transfer (IVF-ET) offers a crucial path to parenthood, with over 8 million children born through this technology globally (5). Yet IVF-ET itself is a major life stressor. Women undergoing treatment commonly experience anxiety, depression, sleep disturbances, and somatic symptoms (6), which have been associated with treatment interruption or discontinuation (7–9). Furthermore, psychological distress has been proposed to activate the hypothalamic–pituitary–adrenal (HPA) axis and may suppress the hypothalamic–pituitary–ovarian (HPO) axis (10). These neuroendocrine changes may adversely affect ovarian stimulation, elevate catecholamine release, and induce uterine contractions, and may be linked to reduced embryo implantation and clinical pregnancy rates.

Prior research has linked specific psychological factors to poorer assisted reproductive outcomes (11, 12). For instance, good self-reported sleep has been associated with higher clinical pregnancy and live birth rates (13), while severe anxiety before treatment has been tied to lower fertilization rates and reduced blastocyst/oocyte and blastocyst/metaphase II ratios (14). A systematic review concluded that infertile women with elevated anxiety or depression are less likely to achieve pregnancy after IVF/ICSI (11). These studies, however, have largely examined symptoms in isolation. In reality, anxiety, depression, and sleep problems in this population commonly co-occur (15) and are thought to be mutually reinforcing within an interconnected symptom network. Identifying the most central and bridging symptoms within this network remains an open question, representing a key evidence gap for future hypothesis-driven psychological intervention research.

Network analysis provides a framework uniquely suited to address this gap. By modeling interactions among individual symptoms rather than relying on aggregate diagnoses (16), it can identify highly connected central symptoms within the distress network and bridge symptoms that link distinct symptom clusters. In this study, we applied network analysis to identify central and bridge symptoms of psychological distress and to explore their associations with IVF-ET outcomes. Our objective was to provide hypothesis-generating evidence for symptom-focused clinical research in this vulnerable population.

## 2 Methods

### 2.1 Setting and population

We conducted a retrospective cohort study at West China Second University Hospital of Sichuan University, a women's and children's medical center located in southwest China. Infertile individuals undergoing their first *in vitro* fertilization (IVF) or intracytoplasmic sperm injection (ICSI) cycle who had completed a psychological assessment at our medical center were included in the study. Eligible participants were required to be 20–45 years old, with no recent history of major psychological trauma, diagnosed psychiatric disorders, or current use of psychotropic medications. Participants who did not start IVF or ICSI treatment after psychological assessment were excluded.

### 2.2 Assessment of psychological exposure

To identify participants with clinically significant psychological distress, a systematic psychological assessment was incorporated into the standard clinical workflow at our institution. The assessment was administered by trained nursing staff to all participants prior to the initiation of ovarian stimulation.

***Anxiety***. The severity of anxiety symptoms was measured using the Generalized Anxiety Disorder-7 (GAD-7) scale. The GAD-7 was originally developed by Spitzer et al. (17), and translated for the Chinese population by He et al. (18). This self-report tool evaluates the frequency of anxiety symptoms over the preceding 2 weeks, whose items each represent a specific symptom. Its seven items are rated on a four-point Likert scale from 0 (not at all) to 3 (nearly every day). The GAD-7 has demonstrated good reliability and validity in previous studies (18), which was supported in the current sample with a Cronbach's α of 0.863. Anxiety was treated as a dichotomous variable, with a total score greater than 4 indicating an abnormal level of anxiety.

***Depression***. Depressive symptoms were assessed with the Patient Health Questionnaire-9 (PHQ-9), developed by Kroenke et al. (19). The PHQ-9 measures the frequency of depression-related symptoms over the past 2 weeks, whose items each represent a specific symptom; its psychometric properties have been established in populations with infertility (20). In this study, the scale achieved a Cronbach's α of 0.812. A dichotomous variable for depression was created, using a cut-off score of >4 to define an abnormal depressive state.

***Somatic symptom***. The Patient Health Questionnaire-15 (PHQ-15), also developed by Kroenke et al. (21), was used to evaluate the severity of somatic symptoms. Somatic symptoms were included in the psychological distress network as physical manifestations of psychological stress, consistent with the biopsychosocial model and the network approach's focus on cross-domain mind-body interactions. This instrument assesses the degree of bother caused by various physical symptoms during the previous 2 weeks, with response options ranging from 0 (not bothered at all) to 2 (bothered a lot). The present study found a Cronbach's α of 0.787 for this scale. Somatic symptoms were dichotomized, with a score exceeding 4 signifying the presence of abnormal somatic symptoms.

***Sleep quality***. The Pittsburgh Sleep Quality Index (PSQI), a self-rated questionnaire was used to assess sleep quality and disturbances over a 1-month interval (22). The PSQI generates seven component scores (e.g., subjective sleep quality, sleep latency, sleep duration), each ranging from 0 to 3. These are summed to produce a global score between 0 and 21. The PSQI has been validated for use in mainland China (13). In this study, it showed a Cronbach's α of 0.742. Subjective sleep quality was analyzed as a dichotomous variable, with a global score of >7 indicating poor sleep quality.

### 2.3 Assessment of IVF-ET outcomes and covariates

Sociodemographic characteristics of study participants (e.g., age, ethnicity, and marital status), medical history (e.g., BMI, infertility duration, infertility type, and gravidity), and laboratory data (e.g., AMH) were extracted from the Health Information System. The characteristics (e.g., age, occupational status, educational level and BMI) of participants' husbands were also collected. All medical information was reviewed, verified, and documented by a qualified physician. The BMI was calculated as weight in kilograms divided by height in meters squared. The IVF-ET outcomes of interest in this study included clinical pregnancy, early miscarriage, late miscarriage, preterm birth, and live birth.

a) Clinical pregnancy was defined as the presence of at least one gestational sac on transvaginal ultrasonography, including intrauterine pregnancy, ectopic pregnancy, and heterotopic pregnancy. Multiple gestational sacs in a single embryo transfer cycle were counted as one clinical pregnancy event. The clinical pregnancy rate was calculated as the number of clinical pregnancies divided by the total number of embryo transfer cycles, expressed as a percentage.
b) Early miscarriage was defined as spontaneous pregnancy loss occurring before 12 completed weeks of gestation among clinically pregnant participants. The early miscarriage rate was calculated as the proportion of clinical pregnancies ending in early miscarriage, expressed as a percentage.
c) Late miscarriage was defined as spontaneous pregnancy loss occurring between 12 completed weeks and before 28 completed weeks of gestation. The late miscarriage rate was calculated as the proportion of clinical pregnancies ending in late miscarriage, expressed as a percentage.
d) Preterm birth was defined as delivery occurring at ≥28 completed weeks and before 37 completed weeks of gestation. The preterm birth rate was calculated as the proportion of live births resulting in preterm delivery, expressed as a percentage.
e) Live birth was defined as the delivery of at least one fetus with detectable signs of life, regardless of gestational age. The live birth rate was calculated as the number of live birth events divided by the total number of embryo transfer cycles, expressed as a percentage.

### 2.4 Data analysis

Data were analyzed using SPSS 26.0 (IBM Corp.) and R version 4.5.1. Normally distributed continuous variables were summarized as mean ± standard deviation, non-normally distributed ones as median and interquartile range, and categorical variables as frequencies and percentages. Diminished ovarian reserve (DOR) was defined as FSH >10 IU/L. Baseline characteristics were compared across enrollment years (2020–2023). The annual incidence of psychological distress was calculated, and differences in prevalence and mean scores were tested using the chi-square test and ANOVA with Bonferroni post-hoc correction, respectively.

The psychological symptom network in this study is rooted in psychopathological network theory, which frames psychological distress as an interconnected system of individual symptoms. Each symptom acts as a node, and the regularized partial correlation between any two symptoms (adjusted for all others) serves as an edge to measure the strength of their association (23). A Gaussian graphical model (GGM) was constructed to examine the symptom network. Because all symptom items were ordinal, polychoric correlations (via cor_auto in *qgraph*) were used for the input matrix. Three items were removed due to conceptual overlap: PHQ-9 item 3 (sleep problems) and PHQ-15 item 15 (sleep problems) both overlapped with the PSQI, while PHQ-15 item 14 (fatigue) overlapped with PHQ-9 item 4 (fatigue). This ensured that each node represented a distinct symptom dimension. The network was estimated using the graphical lasso (GLASSO) in the *qgraph* package, with model selection by the Extended Bayesian Information Criterion (γ = 0.5) to produce a sparse and interpretable network. Edge thickness indicates partial correlation strength, and edge color denotes the direction of association (e.g., blue for positive, red for negative). Node centrality was assessed by expected influence (EI), betweenness, closeness, and predictability (*R*^2^). Predictability was computed using the *mgm* package with 10-fold cross-validation. EI was prioritized owing to its superior stability in psychological networks. Bridge expected influence (BEI) was also computed to identify symptoms that bridge distinct symptom communities. Communities were defined *a priori* by the originating instruments: depression, anxiety, somatic symptoms, and sleep quality. Symptoms with BEI above the 80th percentile were classified as bridge symptoms, and the stability of bridge expected influence was assessed using case-dropping bootstrap. Network stability and accuracy were evaluated via bootstrapping (*bootnet* package): nonparametric bootstrap (1,000 iterations) assessed edge weight accuracy, and case-dropping bootstrap assessed centrality and bridge centrality stability.

To explore whether symptom network characteristics varied by infertility duration, participants were divided into two groups: <5 years (Group 1) and ≥5 years (Group 2). This 5-year cutoff was selected as it represents a conventional clinical threshold for distinguishing short- and long-term infertility, and the same grouping criterion has been adopted in prior related studies on psychological distress in infertile populations (24). Network Comparison Tests (NCT) were used to compare overall network structure, global strength, and edge weights between groups. Differences in individual node centrality and BEI were examined using bootstrapped difference tests. Group differences in BEI were further assessed with Wilcoxon rank-sum tests, and Spearman correlations evaluated the consistency of bridge centrality rankings across groups. Psychological distress was defined as a score above the validated threshold on the relevant scale and was dichotomized accordingly. Clinical pregnancy and other binary outcomes were compared between participants with and without psychological distress using the chi-square test. Multivariable modified Poisson regression with robust error variance was applied to explore the independent associations between subjective sleep quality, sleep medication use, sleep disturbance and outcomes of interest (e.g., clinical pregnancy). Adjusted risk ratios (*aRRs*) with 95% confidence intervals (CIs) were calculated to quantify the strength of associations. In the multivariate Poisson regression analysis, age and BMI were first incorporated and a final model further included occupational status, ethnicity, educational level, FSH, AMH, length of infertility, menses, parity, number of spontaneous abortion and number of induced abortions, and type of embryo transfer.

## 3 Results

### 3.1 Baseline characteristics of participants

A total of 15,642 participants completed psychological assessment comprising four scales: GAD-7, PHQ-9, PHQ-15, and PSQI. Supplementary Figure 1 shows the recruitment, withdrawal and outcome of the participants. The majority of participants were of Han ethnicity, and the largest age group (44.7%) was 30–34 years old (Table 1). The largest infertility duration group (80.2%) was <5 years (Supplementary Table 1). A total of 11,986 participants underwent IVF-ET, with 6,213 in fresh cycles and 5,773 in frozen cycles.

**Table 1:** Characteristics of participants (*n* = 15,642).

Characteristics	All (*n* = 15,642)	2020 (*n* = 3,311)	2021 (*n* = 4,732)	2022 (*n* = 3,673)	2023 (*n* = 3,926)
***Female***					
Age					
<30yr	5,179 (33.1)	1,055 (31.9)	1,604 (33.9)	1,235 (33.6)	1,285 (32.7)
30–34yr	6,987 (44.7)	1,510 (45.6)	2,078 (43.9)	1,631 (44.4)	1,768 (45.0)
35–40yr	2,674 (17.1)	556 (16.8)	804 (17.0)	616 (16.8)	698 (17.8)
40yr and above	802 (5.1)	190 (5.7)	246 (5.2)	191 (5.2)	175 (4.5)
Ethnicity					
Han	14,973 (95.7)	3,153 (95.2)	4,522 (95.6)	3,529 (96.1)	3,769 (96.0)
Minority	669 (4.3)	158 (4.8)	210 (4.4)	144 (3.9)	157 (4.0)
Marital status					
First marriage	13,433 (85.9)	2,847 (86.0)	4,042 (85.4)	3,136 (85.4)	3,408 (86.8)
Remarriage	2,198 (14.0)	464 (14.0)	685 (14.5)	532 (14.5)	517 (13.2)
Data missing	11 (0.1)	0 (0)	5 (0.1)	5 (0.1)	1 (0)
Occupational status					
Employed	13,912 (88.9)	2,908 (87.9)	4,153 (87.8)	3,290 (89.6)	3,561 (90.7)
Not working	1,725 (11.0)	402 (12.1)	575 (12.2)	383 (10.4)	365 (9.3)
Data missing	5 (0.1)	1 (0)	4 (0)	0 (0)	0 (0)
Educational level					
College and below	8,914 (57.0)	2,007 (60.6)	2,783 (58.8)	2,023 (55.1)	2,101 (53.5)
Bachelor	5,563 (35.6)	1,096 (33.1)	1,623 (34.3)	1,347 (36.7)	1,497 (38.1)
Master and above	1,140 (7.3)	207 (6.3)	318 (6.7)	297 (8.1)	318 (8.1)
Data missing	25 (0.1)	1 (0)	8 (0.2)	6 (0.2)	10 (0.3)
***Male***					
Age					
<30yr	3,478 (22.2)	729 (22.0)	1,043 (22.0)	838 (22.8)	868 (22.1)
30–34yr	7,120 (45.5)	1,490 (45.0)	2,170 (45.9)	1,658 (45.1)	1,802 (45.9)
35yr and above	5,044 (32.2)	1,092 (33.0)	1,519 (32.1)	1,177 (32.1)	1,256 (32.0)
Ethnicity					
Han	14,997 (95.9)	3,169 (95.7)	4,524 (95.7)	3,531 (96.2)	3,773 (96.1)
Minority	640 (4.1)	142 (4.3)	205 (4.3)	141 (3.8)	152 (3.9)
Data missing	5 (0)	0 (0)	3 (0)	1 (0)	1 (0)
Marital status					
First marriage	13,749 (87.9)	2,910 (87.9)	4,133 (87.3)	3,217 (87.6)	3,489 (88.9)
Remarriage	1,860 (11.9)	385 (11.6)	585 (12.4)	454 (12.4)	436 (11.1)
Data missing	33 (0.2)	16 (0.5)	14 (0.3)	2 (0)	1 (0)
Occupational status					
Employed	15,150 (96.9)	3,138 (94.8)	4,538 (96.0)	3,614 (98.4)	3,860 (98.3)
Not working	474 (3.0)	170 (5.1)	181 (3.8)	59 (1.6)	64 (1.6)
Data missing	18 (0.1)	3 (0.1)	13 (0.2)	0 (0)	2 (0.1)
Educational level					
College and below	8,684 (55.5)	1,943 (58.7)	2,639 (55.8)	2,019 (55.0)	2,083 (53.1)
Bachelor	5,578 (35.7)	1,101 (33.3)	1,674 (35.4)	1,317 (35.8)	1,486 (37.9)
Master and above	1,346 (8.6)	265 (8.0)	412 (8.7)	331 (9.0)	338 (8.6)
Data missing	34 (0.2)	2 (0)	7 (0.1)	6 (0.2)	19 (0.4)

### 3.2 Prevalence of psychological distress

**Figure 1:**
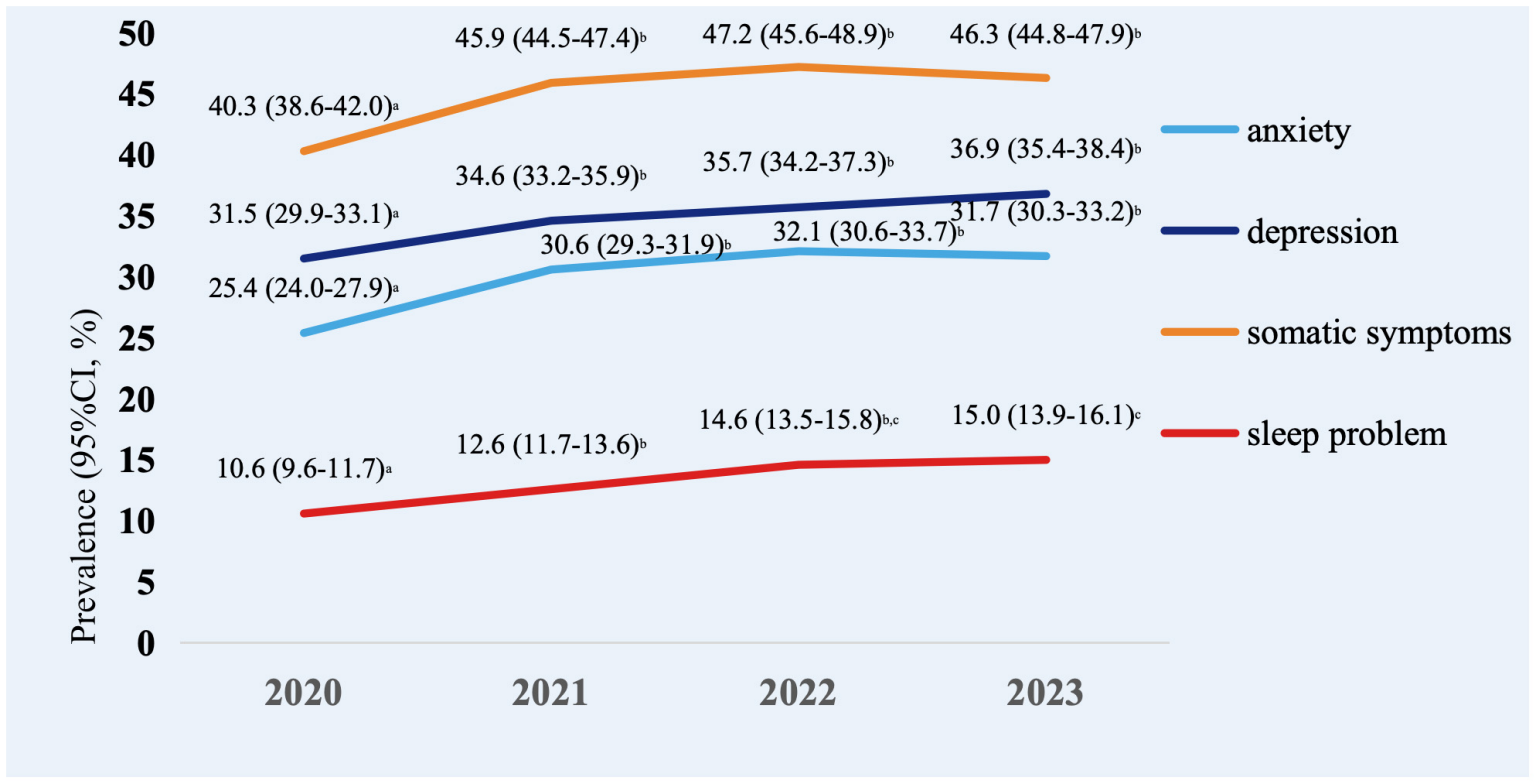
The prevalence of psychological distress (anxiety, depression, somatic symptoms and sleep problems) among participants from 2020 to 2023. : Different superscript letters indicate statistically significant differences).

The prevalence rates of anxiety, depression, somatic symptoms, and sleep problems were 30.1%, 34.8%, 45.1%, and 13.3%, respectively. The mean scores for GAD-7 (*F* = 21.856, *p* < 0.001), PHQ-9 (*F* = 12.690, *p* < 0.001), PHQ-15 (*F* = 12.805, *p* < 0.001), and PSQI (*F* = 27.161, *p* < 0.001) in 2021, 2022, and 2023 were all higher than those in 2020 (Supplementary Figure 2). As shown in Figure 1, the prevalence of anxiety (χ^2^ = 46.679, *p* < 0.001), depression (χ^2^ = 24.969 *p* < 0.001), somatic symptom (χ^2^ = 41.557, *p* < 0.001) and sleep problems (χ^2^ = 37.703, *p* < 0.001) were statistically different in different years. Somatic symptoms had the highest prevalence in 2022 (47.2%). Significant increasing trends from 2020 to 2023 were also identified for several conditions: depression rose from 31.5% to 36.9% (risk difference = 5.4%, 95%CI 3.2%−7.6%) and sleep problems rose from 10.6% to 15.0% (risk difference = 4.4%, 95%CI 2.8%−5.9%). Additionally, the prevalence of anxiety (risk difference = 6.7%, 95%CI 4.6%−8.8%) and somatic symptoms (risk difference = 7.0%, 95%CI 4.7%−9.3%) exhibited a significant increase from 2020 to 2022.

### 3.3 Central symptom and bridging symptom of psychological distress

**Figure 2:**
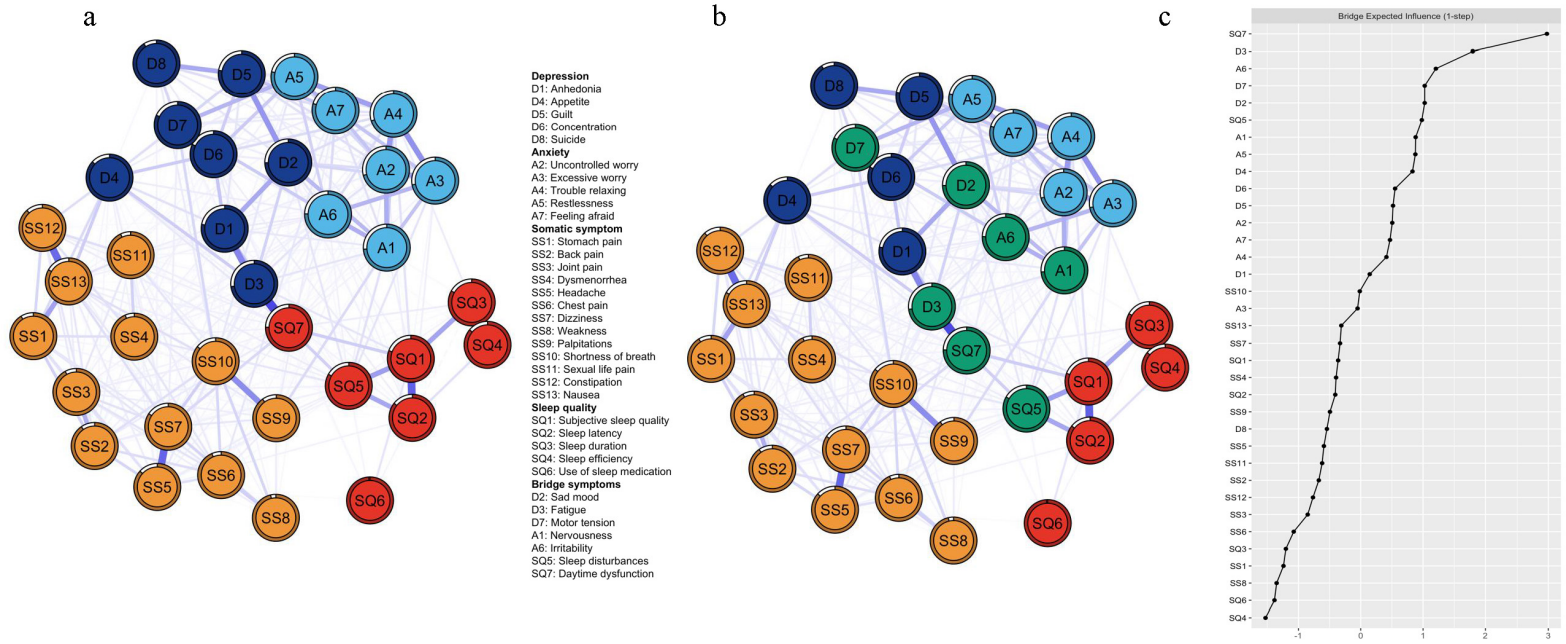
Network structure with node predictability, influence estimates and bridge symptoms (N = 15,642). **(a)** Network structure with nodes interactions and attributes, **(b)** Network structure with 7 bridge symptoms, **(c)** Bridge expected influence (1-step).

The descriptive statistics of the PSQI, GAD-7, PHQ-9, and PHQ-15 items are shown in Supplementary Table 2. The network of anxiety, depression, somatic symptoms, and sleep is shown in Figure 2a. The centrality plot is presented in Supplementary Figure 3. The accuracy and stability are presented in Supplementary Figures 4–9. The results showed that the most central symptoms were trouble relaxing (A4, EI = 1.313), uncontrolled worry (A2, EI = 1.240), daytime dysfunction (SQ7, EI = 1.156), and fatigue (D3, EI = 1.142). The bridge network is shown in Figure 2b, and the centrality plot of bridge expected influence (z-score) is presented in Figure 2c. BEI indices indicated that daytime dysfunction (SQ7, BEI = 0.887), fatigue (D3, BEI = 0.657), irritability (A6, BEI = 0.542), and motor tension (D7, BEI = 0.507) were the strongest bridging symptoms that connected the anxiety, depression, somatic symptom, and sleep disorder communities.

**Figure 3:**
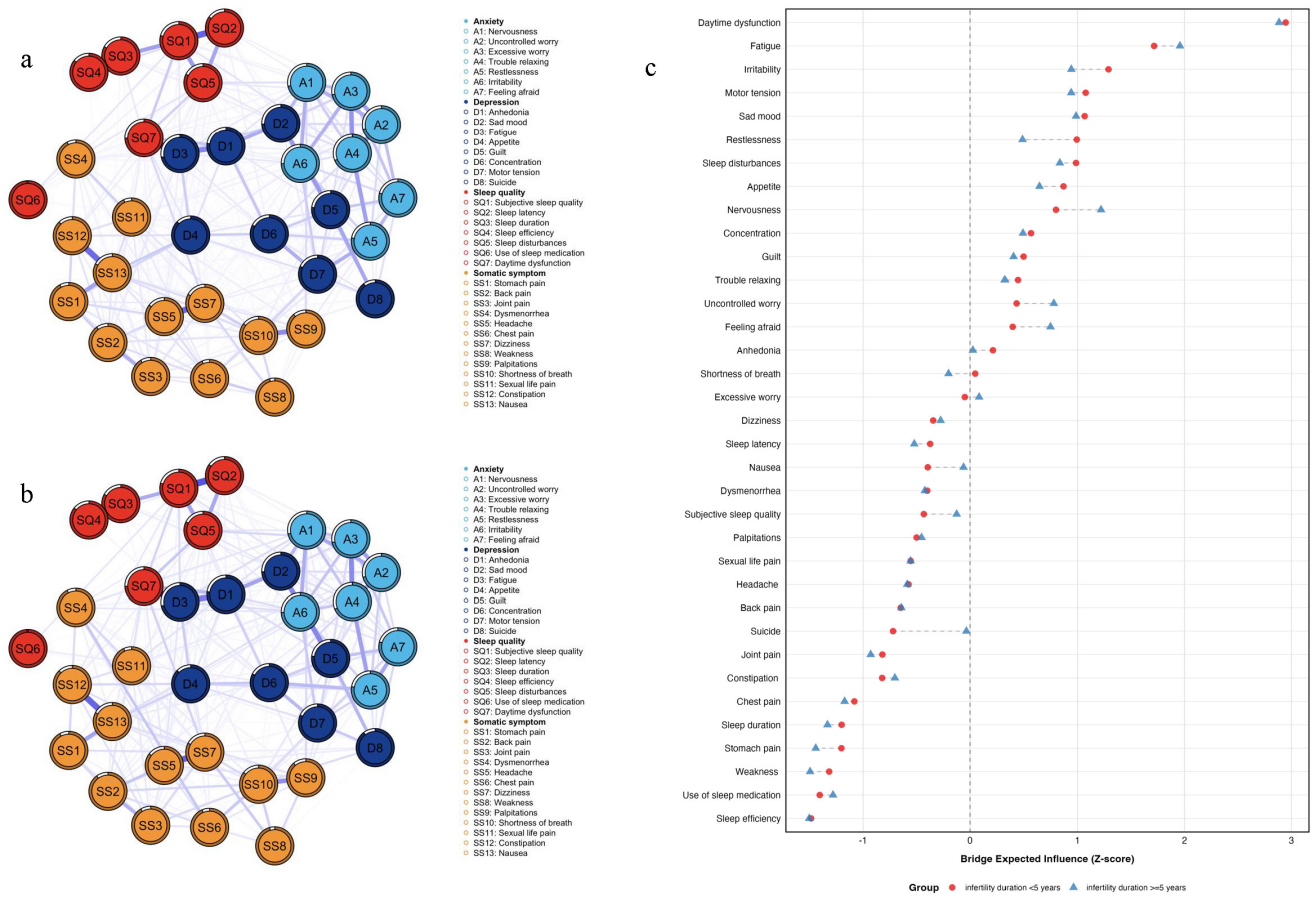
Comparison of network structure and influence by infertility duration. **(a)** Network for infertility duration <5 years (N =12,541), **(b)** Network for infertility duration ≥ 5 years (N = 3,082), **(c)** Bridge expected influence by infertility duration.

We compared the network between the group of infertility duration <5 years (group 1) and the group of infertility duration ≥5 years (group 2). The network comparison of centrality plot is presented in Supplementary Figure 10. There was no statistical difference in network invariance (statistic = 0.07, *p* = 0.38). However, global strength was higher in group 1 than that of group 2 (strength: 14.84 vs. 14.36, statistic = 0.47, *p* =0.04). The ranking of the strongest expected influence nodes in the Group 2 was trouble relaxing (A4), uncontrolled worry (A2), fatigue (D3), and nervousness (A1), which differs from the results of the overall network and Group 1. No statistically significant difference was found in bridge expected influence between the groups (*p* = 0.74). Bridge network comparison showed that the largest differences in symptoms were suicide thoughts (D8, BEI difference_group1 − group2_ = −0.15), nervousness (A1, BEI difference _group1 − group2_ = −0.10), and feeling afraid (A7, BEI difference _group1 − group2_ = −0.09) (Figure 3).

### 3.4 Sleep quality associated with IVF-ET outcomes

Univariate analysis revealed no significant difference in the clinical pregnancy rate, early miscarriage, late miscarriage, preterm birth and live birth between participants with and without anxiety. No significant difference in the above outcomes was observed between participants with and without depression, sleep problems and somatic symptoms (Table 2). Table 3 shows that the clinical pregnancy rate was higher in the group of participants with good subjective sleep quality (χ^2^=, *p* = 0.009). The late miscarriage rate was higher in the group of participants with poor sleep medication use (χ^2^ = 4.959, *p* = 0.026). The total miscarriage rate was higher in the group of participants with poor sleep disturbance (χ^2^ = 4.065, *p* = 0.044). Multivariable modified Poisson regression analysis showed that good subjective sleep quality was associated with a higher likelihood of clinical pregnancy (a*RR* =1.026, 95% CI 1.008–1.044), while good sleep medication use was associated with a lower risk of late miscarriage (a*RR* = 0.211, 95% CI 0.052–0.861) (Table 4).

**Table 2:** Univariate analysis of anxiety, depression, somatic symptoms, and *in vitro* fertilization embryo transfer outcomes.

Outcomes	Anxiety			Depression			Somatic symptoms		
	Positive	Negative	*χ^2^*	Positive	Negative	*χ^2^*	Positive	Negative	*χ^2^*
Clinical pregnancy rate	54.7	54.6	0.008	55.5	54.1	2.207	55.0	54.3	0.617
Early miscarriage rate	10.1	10.9	0.948	10.6	10.7	0.027	10.8	10.6	0.060
Late miscarriage rate	1.3	1.2	0.311	1.1	1.3	0.488	1.2	1.2	0.050
Total miscarriage rate	11.4	12.1	0.547	11.7	12.0	0.156	12.0	11.8	0.025
Preterm birth rate	12.1	13.2	1.229	12.7	12.9	0.042	12.7	12.9	0.082
Live birth rate	45.3	44.6	0.495	45.6	44.4	1.654	45.1	44.5	0.660

**Table 3:** Univariate analysis of sleep quality and *in vitro* fertilization embryo transfer outcomes.

Outcomes	Total score			Subjective sleep quality			Sleep latency			Sleep duration		
	Good	Poor	*χ^2^*	Good	Poor	*χ^2^*	Good	Poor	*χ^2^*	Good	Poor	*χ^2^*
Clinical pregnancy rate	54.8	53.2	1.372	55.1	51.5	6.865^**^	55.1	53.7	2.255	54.8	53.0	1.415
Early miscarriage rate	10.5	11.5	0.738	10.6	11.0	0.125	11.0	10.0	1.703	10.6	11.2	0.261
Late miscarriage rate	1.2	1.4	0.188	1.2	1.3	0.015	1.1	1.3	0.485	1.2	1.2	<0.001
Total miscarriage rate	11.7	12.9	0.934	11.8	12.3	0.143	12.2	11.4	1.016	11.8	12.5	0.232
Preterm birth rate	12.8	13.0	0.009	12.9	12.7	0.016	13.3	12.0	1.937	12.6	15.4	3.485
Live birth rate	45.0	43.3	1.669	45.1	42.7	3.196	45.0	44.6	0.171	45.0	43.4	1.063
**Outcomes**	**Sleep efficiency**			**Sleep disturbances**			**Use of sleep medication**			**Daytime dysfunction**		
	**Good**	**Poor**	*χ^2^*	**Good**	**Poor**	*χ^2^*	**Good**	**Poor**	*χ^2^*	**Good**	**Poor**	*χ^2^*
Clinical pregnancy rate	54.6	56.3	0.193	54.6	53.8	0.126	64.6	57.4	0.207	54.4	55.2	0.518
Early miscarriage rate	10.7	11.1	0.021	10.5	13.9	3.171	10.7	12.8	0.192	10.8	10.3	0.267
Late miscarriage rate	1.2	2.0	0.530	1.2	1.8	0.876	1.2	5.1	4.959^*^	1.3	0.9	1.876
Total miscarriage rate	11.9	13.1	0.149	11.7	15.8	4.065^*^	11.8	17.9	1.377	12.1	11.2	0.918
Preterm birth rate	12.8	15.4	0.455	12.8	14.7	0.668	12.8	13.8	0.023	13.2	11.9	1.475
Live birth rate	44.8	44.3	0.018	45.0	41.6	2.183	44.8	42.6	0.129	44.6	45.6	0.959

^*^p <0.05 ^**^ p <0.01.

**Table 4:** Associations of subjective sleep quality, sleep duration, sleep disturbances and use of sleep medication with *in vitro* fertilization embryo transfer outcomes.

Variables	Crude	Model 1^a^	Model 2^b^
Risk ratio (95%CI)			
Clinical pregnancy rate			
Subjective sleep quality			
Good	1.024 (1.006–1.042)	1.018 (1.001–1.036)	1.026 (1.008–1.044)
Poor	1.00 (ref.)	1.00 (ref.)	1.00 (ref.)
Late miscarriage rate			
Sleep medication use			
Good	0.223 (0.056–0.887)	0.215 (0.054–0.862)	0.211 (0.052–0.861)
Poor	1.00 (ref.)	1.00 (ref.)	1.00 (ref.)
Total miscarriage rate			
Sleep disturbances			
Good	0.755 (0.562–1.013)	0.762 (0.567–1.023)	0.753 (0.561–1.010)
Poor	1.00 (ref.)	1.00 (ref.)	1.00 (ref.)

^a^Adjusted for age and body mass index; ^b^Adjusted for occupational status, ethnicity, educational level, FSH, AMH, length of infertility, menses, parity, number of spontaneous abortions and induced abortions, type of embryo transfer.

## 4 Discussion

This study used network analysis to map the symptom network of anxiety, depression, somatic symptoms, and sleep problems in infertile women. We found that somatic symptoms were the most common form of psychological distress. Highly connected central symptoms among participants included trouble relaxing, uncontrolled worry, daytime dysfunction, and fatigue. Daytime dysfunction, fatigue, and irritability served as key bridge nodes linking symptoms across different symptom communities. Specific domains of sleep quality were associated with IVF-ET outcomes, such as clinical pregnancy and miscarriage. Therefore, sleep-related symptoms may provide hypothesis-generating clues for future clinical intervention research in this population.

Our study revealed a high and increasing burden of psychological distress in infertile women, with somatic symptoms being the most common (45.1%), followed by depression (34.8%), anxiety (30.1%), and sleep problems (13.8%). The prevalence of anxiety and depression in our cohort is consistent with a previous meta-analysis of 40 studies (*n* = 16,042), which reported pooled rates of 48.0% for anxiety and 35.6% for depression in infertile women (15). Differences in prevalence across studies can be attributed to variations in country income level, continent, assessment tools, and sample size. In addition, the statistically significant upward trend in all four psychological distress indicators from 2020 to 2023 is a concerning finding, supported by Liu's finding (25). That systematic review of 19,480 participants used a comparative design and found that the pooled prevalence of anxiety in the pandemic group (40.3%) was significantly higher than that in the control group (27.3%). One explanation for this trend is the protracted impact of the COVID-19 pandemic. Social distancing, disruptions to medical care, and pervasive health anxieties during this period likely added to the psychological burden of this vulnerable population (26). The high prevalence of somatic symptoms suggests that in our cultural context, psychological distress is frequently manifested through somatic expressions. This finding is consistent with Pei's (27) observation of moderate distress disclosure, suggesting that inhibition of direct emotional expression may be a key mechanism underlying this somatic pathway. The specific stresses of infertility, including anxiety about conception and the ordeal of unsuccessful treatments, are closely associated with physical symptoms such as fatigue. This interpretation aligns with our symptom network, which identified fatigue as a central and bridging symptom. Collectively, these findings further support that psychological distress is a widespread and significant concern for infertile women globally. These findings suggest that routine psychological screening covering both emotional and somatic symptoms may have clinical relevance and could be considered in reproductive medical setting. More importantly, psychological distress, particularly anxiety, has been identified as a factor associated with infertility (a*OR*: 9.99; 95%CI 2.86–34.92) (12). The growing prevalence of psychological distress also highlights the potential value of targeted symptom-focused interventions, which remains to be validated by further interventional research. From a health policy perspective, these findings provide preliminary evidence supporting the integration of psychological services into routine infertility care, and further health services research is warranted to guide resource allocation.

Our network analysis found that trouble relaxing and uncontrolled worry were the most central symptoms. They had the highest expected influence (EI) in the psychological network of infertile women. This matches previous research showing that anxiety, especially generalized anxiety, is often the main form of psychological distress in infertility (15). Uncertainty about treatment outcomes and a lack of perceived control over the future may contribute to these persistent anxious feelings (28). Fatigue (D3) and daytime dysfunction (SQ7) were not only central symptoms but also strong bridge symptoms. This finding aligns with a potential role of mind-body interaction in infertility-related distress. Emotional distress and sleep problems appear to be mutually reinforcing in association with fatigue, a pattern that aligns with the common experience of combined psychological and physical exhaustion among patients. Bridge symptoms may represent highly connected nodes associated with the co-occurrence of comorbid mental symptoms. These findings generate the hypothesis that treatments targeting these symptoms could be effective, although this awaits empirical testing in intervention studies. Daytime dysfunction is classified under sleep disorders because it is a core part of the PSQI. But it also emerged as a strong bridge symptom connecting to somatic symptoms. This makes sense. The fatigue, low energy, and functional impairment of daytime dysfunction directly overlap with and are closely linked to more severe somatic symptoms. Its bridging role suggests that sleep status is closely associated with both nighttime sleep experience and daytime emotional and physical wellbeing. These findings suggested that symptoms like trouble relaxing, uncontrolled worry, fatigue, and daytime dysfunction are not just common complaints. They occupy highly central structural positions in the psychological distress network. Elevations in these symptoms are closely associated with concurrent elevations in other symptoms (e.g., anhedonia, excessive worry, and guilt), with associations spanning across psychological domains. Given this pattern, the network results generate the hypothesis that psychological interventions targeting these high-centrality and bridge symptoms are potential clinical priorities; future intervention research is needed to directly test this possibility. A qualitative systematic review reported that infertile women need accessible psychological interventions to reduce emotional distress (e.g., relaxation and stress release) related to infertility and ART (29). Another systematic review found that cognitive behavioral therapy and counseling were among the most effective approaches for anxiety and depression in women with infertility (30). Mindfulness-based therapy may also be theoretically suitable for addressing core symptoms such as uncontrolled worry and trouble relaxing. It teaches focused attention to break ruminative cycles and acceptance to reduce cognitive struggle, all without medication (31).

Network comparison showed that infertility duration was associated with difference in the global strength of the psychological network but not its underlying structure, as well as shifts in the relative importance of core symptoms. Regardless of infertility duration, the basic interaction patterns among anxiety, depression, somatic symptoms, and sleep problems remained similar, suggesting a common core psychopathological process in infertile women. However, group 1 (<5 years) had significantly greater global strength, indicating that in the early stage of fertility challenges, symptoms exhibited stronger interconnections with each other, forming a more tightly interconnected symptom network. In contrast, the prolonged duration group (≥5 years) may be accompanied by a form of maladaptive psychological adjustment, which could correspond to the observed slight reduction connectivity. This pattern aligns with a model of cumulative stress (32). The higher connectivity in the short-duration group may correspond to an acute phase of heightened psychological distress. The lower connectivity in the long-duration group may represent maladaptive exhaustion or emotional numbing, a proposed psychological adaptation to chronic stress, consistent with evidence linking longer infertility duration to higher levels of depression and infertility-related stress (20, 33). In the prolonged duration group, nervousness (A1) ranked among the most central symptoms. This may indicate that as infertility persists, persistent background nervousness may become more prominent relative to acute worry. Notably, no statistically significant between-group difference was detected in bridge expected influence (BEI). The positive BEI value of suicidal ideation was only numerically higher in the prolonged group, reflecting a weak descriptive trend rather than a robust difference. This exploratory finding must be interpreted with extreme caution: it does not imply a higher prevalence or severity of suicidal ideation, but only a potential shift in its correlational patterns with other distress symptoms. As a purely hypothesis-generating observation requiring further prospective validation, clinicians may consider integrating routine suicidal ideation screening into psychological assessment for patients with prolonged infertility, even in the absence of severe depressive symptoms.

Beyond mapping the psychological symptom network, our study identified specific sleep-related factors associated with IVF-ET outcomes. We found that poor subjective sleep quality was negatively associated with clinical pregnancy, consistent with previous studies (34). One prospective study reported that women with good sleep quality had a clinical pregnancy rate of 69.3% vs. 65.1% for those with poor sleep quality, and a live birth rate of 50.5% vs. 45.7% (13). The association between poor sleep quality and clinical pregnancy may be explained by disruption of hormonal axes (35). A cross-sectional study of 1,070 women aged 20–40 years using the PSQI and ovarian reserve biomarkers showed that women with poor sleep had significantly lower AMH and AFC levels and higher FSH levels than those with better sleep (36). A prospective cohort study of 174 women who completed the PSQI before IVF/ICSI observed a 22% reduction in total and mature oocytes retrieved in those reporting fairly bad or very bad sleep, after adjusting for age, BMI and parity (37). Additionally, we observed that sleep medication use showed a tentative positive association with late miscarriage rate; however, this finding must be interpreted with great caution. Among the 170 participants who reported using sleep medication, only three experienced late miscarriage, and the corresponding confidence interval was wide, indicating substantial imprecision. Given the small number of events, this association is exploratory and cannot support any causal inference. Several potential biological mechanisms have been proposed in the literature to explain this possible association, should it be replicated in future studies. First, sleep medication and untreated sleep disturbances (e.g., insomnia) may dysregulate the HPA axis and raise cortisol levels (38). Chronically elevated cortisol has been associated with impaired placental function and increase uterine artery resistance, which may threaten fetal viability, especially in later gestation (39). Second, sleep disturbances have been linked to elevated systemic inflammation and oxidative stress (40), both of which have been associated with embryo implantation failure and early miscarriage (41). It has also been suggested that some medications may directly affect progesterone or other hormonal milieus needed to maintain pregnancy beyond the first trimester. However, given the observational design and the limited number of events, these biological considerations remain speculative. Unmeasured confounding factors, such as the severity of the underlying sleep disorder prompting medication use, might also account for the observed elevation in miscarriage risk. Thus, this finding should be viewed solely as hypothesis-generating and requires replication in larger studies with adequate statistical power.

This study has several limitations: First, as the network analysis was based on cross-sectional data, the estimated connections reflect associations between symptoms at one time point. Although this allows us to identify highly connected central symptoms, it does not allow us to determine causal or temporal relationships between them. Future studies should use longitudinal designs to better understand how symptom networks evolve over time. Secondly, sleep was measured using self-report scales rather than objective tools such as actigraphy, which may have introduced measurement bias. Further research is also needed on the safety of sleep medications during the peri-implantation and early pregnancy stages. Third, our study has limitations in confounding factor adjustment. Several key IVF-related covariates (including stimulation protocol, infertility diagnosis, embryo quality, fresh/frozen transfer status, endometrial thickness, number of retrieved oocytes, etc.) were not fully adjusted in the regression models, due to incomplete medical records and missing data inherent to the retrospective study design. This may introduce residual confounding bias to the effect estimates, and our findings should be interpreted with caution.

## 5 Conclusions

Our findings identified trouble relaxing and uncontrolled worry as the most highly connected central symptoms within the network, suggesting that anxious cognition may play a role in the psychopathology of this population. Fatigue and daytime dysfunction served as both central and bridge symptoms, consistent with a potential role of mind-body interaction in psychological distress. Sleep was also found to be associated with pregnancy outcomes, providing hypothesis-generating support for the potential relevance of sleep health assessment in routine infertility care. Future longitudinal studies with objective measures are needed to verify causal relationships and to evaluate intervention effectiveness.

## Data Availability

The original contributions presented in the study are included in the article/Supplementary material, further inquiries can be directed to the corresponding author.
