# Supplementary material for: Mapping the symptom network of anxiety, depression, somatic symptoms and sleep disturbances in 15,642 infertile women: a retrospective cohort study identifying targets for fertility care
Source: Front Public Health. 2026 Aug 5;14:1884236. doi: 10.3389/fpubh.2026.1884236 (PMC13485595; doi:10.3389/fpubh.2026.1884236)
Supplement: Supplementary file 1 [file Supplementary_file_1.docx]

***Supplementary materials***

[Supplementary Table 1 Assisted reproductive technology related characteristics of participants.	2](#_Toc233098413)

[Supplementary Figure 1 Participant flow diagram	4](#_Toc233098414)

[Supplementary Figure 2 The mean score of GAD-7, PHQ-9, PHQ-15 and PSQI between 2020 and 2023.	5](#_Toc233098415)

[Supplementary Table 2 Descriptive statistics of the PSQI, GAD-7, PHQ-9 and PHQ-15 items.	6](#_Toc233098416)

[Supplementary Table 3 List of nodes, predictability, and centrality estimation.	7](#_Toc233098417)

[Supplementary Figure 3 centrality plot (betweenness, closeness, expected influence) ordered by expected influence.	8](#_Toc233098418)

[Supplementary Figure 4 Accuracy analysis of the edge weights.	9](#_Toc233098419)

[Supplementary Figure 5 Stability of node betweenness, closeness, expected influence, and strength.	10](#_Toc233098420)

[Supplementary Figure 6 Stability of bridge expected influence	11](#_Toc233098421)

[Supplementary Figure 7 Bootstrapped difference test of edge weights.	12](#_Toc233098422)

[Supplementary Figure 8 Bootstrapped difference test of the node expected influence centrality.	13](#_Toc233098423)

[Supplementary Figure 9 Bootstrapped difference test of the bridge expected influence centrality.	14](#_Toc233098424)

[Supplementary Figure 10 Network comparison of centrality plot.	15](#_Toc233098425)

# Supplementary Table 1 Assisted reproductive technology related characteristics of participants.

| Characteristics | All  (n=15642) | 2020 (n=3311) | 2021 (n=4732) | 2022  (n=3673) | 2023 (n=3926) |
| --- | --- | --- | --- | --- | --- |
| BMI (female, kg/m^2^)  <18.5  18.5-23.9  24-27.9  >=28  Data missing  BMI (male, kg/m^2^)  <18.5  18.5-23.9  24-27.9  >=28  Data missing  FSH (IU/L)  <=10  >10  Data missing  AMH (ng/ml)  >1.1  0.5-1.1  <0.5  Data missing  Infertility duration  <5y  5-10y  10y and above  Data missing  Infertility type  Primary  Secondary  Other  Data missing  Gravidity  0  1-2  3 and above  Data missing  Previous delivery  Spontaneous abortion  0  1-2  3 and above  Data missing  Artificial abortion | 1517 (9.7)  10740 (68.7)  2785 (17.8)  573 (3.7)  27 (0.2)  449 (2.9)  6957 (44.5)  6181 (39.5)  2018 (12.9)  37 (0.2)  13332 (85.2)  2066 (13.2)  244 (1.6)  12877 (82.3)  1654 (10.6)  981 (6.3)  130 (0.8)  12541 (80.2)  2585 (16.5)  497 (3.2)  19 (0.1)  8004 (51.2)  5754 (36.8)  1866 (11.9)  18 (0.1)  8684 (55.5)  5169 (33.0)  1782 (11.4)  7 (0.1)  1749 (11.2)  12665 (81.0)  2547 (16.3)  423 (2.7)  7 (0)  2913 (18.6) | 342 (10.3)  2299 (69.4)  545 (16.5)  118 (3.6)  7 (0.2)  120 (3.6)  1494 (45.1)  1287 (38.9)  400 (12.1)  10 (0.3)  2820 (85.2)  437 (13.2)  54 (1.6)  2620 (79.1)  394 (11.9)  283 (8.5)  14 (0.4)  2567 (77.5)  624 (18.8)  116 (3.5)  4 (0.1)  1687 (51.0)  1305 (39.4)  315 (9.5)  4 (0.1)  1843 (55.7)  1074 (32.4)  391 (11.8)  3 (0.1)  389 (11.8)  2708 (81.8)  507 (15.3)  93 (2.8)  3 (0.1)  659 (19.9) | 450 (9.5)  3219 (68.0)  886 (18.7)  167 (3.5)  10 (0.2)  129 (2.7)  2149 (45.4)  1853 (39.2)  587 (12.4)  14 (0.3)  4033 (85.2)  629 (13.3)  70 (1.5)  3803 (80.4)  583 (12.3)  314 (6.6)  32 (0.7)  3735 (78.9)  837 (17.7)  153 (3.2)  7 (0.2)  2339 (49.4)  1798 (38.0)  589 (12.4)  6 (0.2)  2559 (54.1)  1582 (33.4) 589 (12.4)  2 (0.1)  553 (11.7)  3797 (80.2)  789 (16.7)  144 (3.0)  2 (0.1)  904 (19.1) | 354 (9.6)  2511 (68.4)  667 (18.2)  132 (3.6)  9 (0.2)  96 (2.6)  1604 (43.7)  1486 (40.5)  476 (13.0)  11 (0.3)  3130 (85.2)  470 (12.8)  73 (2.0)  3066 (83.5)  363 (9.9)  189 (5.1)  55 (1.5)  2974 (81.0)  573 (15.6)  119 (3.2)  7 (0.2)  1877 (51.1)  1317 (35.9)  472 (12.9)  7 (0.2)  2048 (55.8)  1243 (33.8)  381 (10.4)  1 (0)  411 (11.2)  2997 (81.6)  585 (15.9)  90 (2.5)  1 (0)  672 (18.3) | 371 (9.5)  2711 (69.0)  687 (17.5)  156 (4.0)  1 (0)  104 (2.6)  1710 (43.6)  1555 (39.6)  555 (14.1)  2(0.1)  3349 (85.3)  530 (13.5)  47 91.2)  3388 (86.3)  314 (8.0)  195 (5.0)  29 (0.7)  3265 (83.2)  551 (14.0)  109 (2.8)  1 (0)  2101 (53.5)  1334 (34.0)  490 (12.5)  1 (0)  2234 (56.9)  1270 (32.4)  421 (10.7)  1 (0)  396 (10.1)  3163 (80.6)  666 (17.0)  96 (2.4)  1 (0)  678 (17.3) |

# Supplementary Figure 1 Participant flow diagram.

(ET=embryo transfer)

# Supplementary Figure 2 The mean score of GAD-7, PHQ-9, PHQ-15 and PSQI between 2020 and 2023.

(a. anxiety; b. depression; c. somatic symptom; d. sleep quality; ****P<0.001; The mean difference (95%CI) was used to describe the differences between the two groups)

# Supplementary Table 2 Descriptive statistics of the PSQI, GAD-7, PHQ-9 and PHQ-15 items.

| Node name | Item content | Mean | SD |
| --- | --- | --- | --- |
| **PSQI**  SQ1  SQ2  SQ3  SQ4  SQ5  SQ6  SQ7  **PHQ-15**  SS1  SS2  SS3  SS4  SS5  SS6  SS7  SS8  SS9  SS10  SS11  SS12  SS13  **GAD-7**  A1  A2  A3  A4  A5  A6  A7  **PHQ-9**  D1  D2  D3  D4  D5  D6  D7  D8 | Subjective sleep quality  Sleep latency  Sleep duration  Sleep efficiency  Sleep disturbances  Use of sleeping medications  Daytime dysfunction  Stomach pain  Back pain  Joint pain  Dysmenorrhea Headache  Chest pain  Dizziness  Weakness  Palpitations  Shortness of breath  Sexual life pain  Constipation or diarrhea  Nausea  Nervousness  Uncontrolled worry  Excessive worry  Trouble relaxing  Restlessness  Irritability  Feeling afraid  Anhedonia  Sad mood  Fatigue  Appetite  Guilt  Concentration  Motor tension  Suicide thoughts | **4.84**  0.90  1.33  0.66  0.13  0.78  0.03  1.01  **4.75**  0.26  0.32  0.27  0.68  0.31  0.15  0.28  0.04  0.25  0.16  0.29  0.50  0.34  **3.34**  0.64  0.46  0.67  0.42  0.20  0.66  0.27  **3.80**  0.65  0.55  0.64  0.42  0.35  0.32  0.22  0.07 | **2.47**  0.62  0.83  0.71 0.40  0.52  0.22  0.80  **3.66**  0.47  0.51  0.47  0.61  0.50  0.37  0.48  0.21  0.45  0.38  0.48  0.57  0.51  **3.03**  0.58  0.61  0.64  0.61  0.46  0.65  0.51  **3.14**  0.59  0.57  0.59  0.59  0.56  0.56  0.48  0.29 |

# Supplementary Table 3 List of nodes, predictability, and centrality estimation.

| Node | Expected influence | Bridge expected influence  (1-step) | Predictability (*R^2^*) |
| --- | --- | --- | --- |
| SQ1  SQ2  SQ3  SQ4  SQ5  SQ6  SQ7  SS1  SS2  SS3  SS4  SS5  SS6  SS7  SS8  SS9  SS10  SS11  SS12  SS13  A1  A2  A3  A4  A5  A6  A7  D1  D2  D3  D4  D5  D6  D7  D8 | 0.657  -0.483  -0.447  -1.344  0.491  -3.037  1.156  -0.690  -0.272  -0.622  -1.070  0.051  -0.873  -1.965  -1.965  -0.622  0.629  -0.926  -0.481  0.956  0.861  1.240  0.793  1.313  0.522  1.022  0.298  0.353  0.750  1.142  -0.082  0.688  0.348  0.329  -1.357 | 0.236  0.227  0.072  0.009  0.498  0.037  0.887  0.186  0.175  0.141  0.229  0.191  0.097  0.242  0.043  0.210  0.034  0.186  0.157  0.246  0.478  0.405  0.297  0.387  0.477  0.542  0.398  0.335  0.507  0.657  0.483  0.407  0.414  0.507  0.201 | 0.349  0.265  0.308  0.259  0.306  0.016  0.431  0.169  0.183  0.152  0.124  0.246  0.125  0.288  0.07  0.189  0.264  0.135  0.217  0.306  0.454  0.494  0.480  0.511  0.384  0.431  0.366  0.392  0.440  0.466  0.245  0.397  0.329  0.330  0.173 |

# Supplementary Figure 3 centrality plot (betweenness, closeness, expected influence) ordered by expected influence.

# Supplementary Figure 4 Accuracy analysis of the edge weights.

# Supplementary Figure 5 Stability of node betweenness, closeness, expected influence, and strength.

# Supplementary Figure 6 Stability of bridge expected influence

# Supplementary Figure 7 Bootstrapped difference test of edge weights.

# Supplementary Figure 8 Bootstrapped difference test of the node expected influence centrality.

# Supplementary Figure 9 Bootstrapped difference test of the bridge expected influence centrality.

# Supplementary Figure 10 Network comparison of centrality plot.
